# Trends and risk factors of hyperglycemia and diabetes among Kuwaiti adults: National Nutrition Surveillance Data from 2002 to 2009

**DOI:** 10.1186/1471-2458-13-103

**Published:** 2013-02-05

**Authors:** Faruk Ahmed, Carol Waslien, Mona A Al-Sumaie, Prasanna Prakash, Ahmad Allafi

**Affiliations:** 1 Nutrition and Dietetics, School of Public Health and Griffith Health Institute, Griffith University, Gold Coast Campus, Queensland, 4222, Australia; 2 Middle East Monitor, Washington, DC, USA; 3 Community Nutrition Promotion Department, Food and Nutrition Administration, Ministry of Health, Safat, Kuwait; 4 Department of Family Sciences, College for Women, Kuwait University, Safat, Kuwait

## Abstract

**Background:**

Current prevalence estimates for diabetes in Arabian Gulf countries are some of the world’s highest, yet regional trends and contributing factors are poorly documented. The present study was designed to determine temporal changes in the prevalence of impaired fasting glucose (IFG) and diabetes and associated factors in Kuwaiti adults.

**Methods:**

Data analysis from the nationally representative cross-sectional Kuwait National Nutrition Surveillance System. 2745 males and 3611 females, aged 20–69 years, attending registration for employment or pensions and Hajj Pilgrimage health check-ups or accompanying children for immunizations from 2002 through 2009 were participated. Socio-demographic and lifestyle information, height and weight, and blood samples were collected.

**Results:**

During the 8 years (2002–09), prevalences of IFG in males and females decreased by 7.4% and 6.8% and of diabetes by 9.8% and 8.9% in males and females, respectively. Linear regression for blood glucose level with time, adjusted for age, BMI, blood cholesterol and education level, showed a greater decrease in males than females (1.12 vs 0.93 mmol/L); males also showed an increase in 2002–2003 followed by a marked decrease in 2006–2007 while females showed a significant decrease in 2008–2009. Both males and females showed the largest decrease in the 2^nd^ half of the study accounting for the majority of the overall decrease (1.13 mmol/L for males and 0.87 mmol/l for females for the 4 years). Compared with 2002–03, the OR for IFG in males decreased with time, and becoming significantly lower (OR=0.32; 95% CI: 0.21-0.49) for 2008–09. In females, the OR for IFG decreased significantly with time, except in 2006–07. Similarly, the OR for diabetes in males decreased to 0.34 (95% CI: 0.24-0.49) and in females to 0.33 (95% CI: 0.22-0.50) in 2008–09. For both genders, age and BMI were independently positively associated with IFG and diabetes, while education levels and smoking were negatively associated with IFG and diabetes. No significant association was found for either hypercholesterolemia or exercise in either gender.

**Conclusion:**

Continued monitoring of blood glucose is needed to see if negative trends observed in 2008–2009 endure and further research of contributing factors is required for development of targeted intervention strategies.

## Background

Hyperglycemia and diabetes prevalences are increasing at an epidemic rate in many parts of the world, with worldwide diabetes prevalence among adults aged 20–79 years estimated to be 6.4% in 2010 and increasing to 7.7% by 2030 [1]. Diabetes is particularly prevalent in the Arabian Gulf countries; Kuwait is in the top ten countries in prevalence of diabetes [1] and diabetes accounts for an increasing number of hospital admissions [2].

This high level of diabetes is a major public health concern as it is associated with increased risk for micro- and macro-vascular complications, and of increased risk of premature death as a consequence [3-5]. Impaired glucose tolerance (IGT) and impaired fasting glucose (IFG) are independent risk factors for diabetes [6]. In 2004, the WHO estimated that 3.4 million people globally died from consequences of high blood glucose levels [7].

One indirect factor contributing to the rise in diabetes prevalence in Arabian Gulf countries may be the increased wealth of the oil-producing countries including Kuwait as the timing of increased wealth is associated with a rapid increase in the prevalence of obesity, diabetes mellitus and cardiovascular diseases [8]. Several studies made an attempt to identify more direct risk factors for diabetes among Kuwaiti populations; however, all were based on small and selected samples [9-12]. Further, only a few took into account potential confounding factors while examining these known risk factors [12]. In addition, there are no reports of temporal changes in the prevalence of diabetes and/or hyperglycemia among the Kuwaiti population. Kuwait instituted the Kuwait National Nutrition Surveillance (KNNS) in 1998 to monitor changes in obesity and biochemical indicators of chronic diseases including blood glucose levels. The present study was carried out to determine 8 year trends in prevalence of hyperglycemia and diabetes among Kuwaiti adults since 2002, and to examine various socio-demographic, biological and lifestyle factors that may have contributed to variation in this prevalence.

## Methods

### Study design and population

A total of 6356 adults (2745 males and 3611 females) ages 20 to 69 years from the KNSS for years 2002 through 2009 have been analyzed. The Administration for Food and Nutrition, Ministry of Health in Kuwait established the KNSS to continuously monitor the nutritional health status of the Kuwaiti population using a serial cross-sectional design. Adults attending mandatory health examinations for registration for government employment or pensions (note: all Kuwaiti citizens have virtually guaranteed employment and retirement pensions and, according to the Public Authority for Civil Information, 80% of employed Kuwaiti men and women worked in the public sector in 2009) or attending mandatory Hajj Pilgrimage health check-ups or accompanying children for mandatory immunizations at local health centers were selected. Informed consent was sought from each participant before data were collected. Refusal rates were less than 5%. Pregnant women or with diagnosed diabetes were excluded from the study. The study was approved by the Ministry of Health, Kuwait.

Surveillance efforts began in 1998 with an assessment of body weight, height and biochemical variables including blood glucose (BG) together with information on age, gender and education level. However, information on fasting or non-fasting status during blood collection was available only from late 2001. Subsequently, information on smoking and exercise were added in 2003. Education level was determined by asking whether they were illiterate, or completed either primary, intermediary, secondary, diploma, undergraduate or postgraduate levels. As Kuwaitis are generally engaged in office-work involving only sedentary activities and do little household work, physical activity was defined as deliberate non-work related exercise outside the home, such as walking, running, or cycling. Those who smoked cigarettes, sheesha (water-pipe) and/or cigars at least once a day were defined as smokers.

### Data collection

Weight and height of subjects was measured in light clothing without shoes. Height was measured to the nearest 0.1 cm, using a stationary height board calibrated in cm. Body weight was measured to the nearest 0.1 kg using a Seca electronic balance (Seca 770, Leicester, UK).

### Biochemical analysis

Finger prick blood samples were analyzed for BG levels using an Accutrend GCT/Accutrend Alpha (Roche Diagnostics GmbH, Mannheim, Germany). Serum total cholesterol (TC) was measured using the Reflotron (Boehringer- Mannheim, Mannheim, Germany).

### Statistical analysis

Normal BG was defined as a fasting blood glucose (FBG) level below 6.1 mmol/L. FBG of 6.1-6.9 mmol/L was considered as IFG and ≥7.0 mmol/L as diabetes [13]. Hypercholesterolemia (HC) was classified as moderate (TC 5.2 – 6.22 mmol/L) and high (TC > 6.22 mmol/L) [14]. Cut-offs used to define overweight (BMI>25.0-29.9 kg/m^2^) and obesity (BMI ≥30.0 kg/m^2^) were taken from the World Health Organization [15]. Chi-square test was performed for males and females separately to detect overall differences in the prevalence of IFG and diabetes with socio-demographic, biological and lifestyle factors. Then differences between sub-groups for each of the variables were examined by Z-test. As the prevalences of both IFG and diabetes in females were significantly (*P*<0.001) lower than males, multivariate analyses were performed males and females separately.

Trends in absolute FBG levels were examined using multiple linear regression analysis with inclusion of year of study, age, BMI, TC and education level, while trends in prevalence of IFG and diabetes were examined using multiple logistic regression analysis by four 2-year time periods compared with 2002–03 for males and females separately using both unadjusted model and after adjustment for age, BMI, HC and education level categories.

A second model of logistic regression was used to identify the risk factors associated with IFG and diabetes in males and females separately by combining all study years with age, BMI, HC, education level, exercise and smoking categories included as independent variables. All statistical analysis was done using SPSS for Windows (version 17 SPSS incorporation, Chicago, IL, USA).

## Results

Table 1 shows the distribution of study participants by various socio-demographic, biological and lifestyle variables within study periods. Overall, females constituted a significantly higher proportion than males in all study period. For both males and females, younger people (20–29 y) constituted the highest proportion (males: 32.2 - 36.2% & females: 29.7 - 39.5%) and older people (60–69 y) the lowest proportion (males: 4.3 - 12.6% & females: 3.6 - 5.3%) in all study periods. Across study periods the prevalence of obesity varied from 31.7- 37.3% in males and 44.0-52.1% in females, of high-TC from 5.0-17.3% in males and 6.7-16.2% in females, of lower education level from 22.1-26.9% in males and 30.5-35.1% in females. Relatively more males (34.4 - 44.6%) were reported to exercise than females (19.2 - 28.6%) and only a small number of females were smokers (0.9 - 5.5%) compared to males (18.1 - 31.3%) across all study periods.

**Table 1:** Characteristics of study participants by study period

	**2002–03**				**2004–05**				**2006–07**				**2008–09**			
	**Male**		**Female**		**Male**		**Female**		**Male**		**Female**		**Male**		**Female**	
	**n**	**%**	**n**	**%**	**n**	**%**	**n**	**%**	**n**	**%**	**n**	**%**	**n**	**%**	**n**	**%**
**Gender**	932	44.1	1181	55.9	579	42.3	790	57.7	716	41.7	1003	58.3	518	44.8	637	55.2
**Age Group (Year)**																
20-29	326	35.0	467	39.5	186	32.2	235	29.7	231	32.3	315	31.4	187	36.2	229	35.9
30-39	221	23.7	192	16.3	138	23.8	214	27.1	178	24.9	279	27.8	123	23.7	172	27.1
40-49	244	26.2	317	26.8	99	17.1	160	20.3	179	25.0	233	23.2	113	21.8	132	20.7
50-59	98	10.5	150	12.7	83	14.3	139	17.6	97	13.5	138	13.8	67	12.9	81	12.7
60-69	43	4.6	55	4.7	73	12.6	42	5.3	31	4.3	38	3.8	28	5.4	23	3.6
**BMI Status**																
Normal (<25 kg/m^2^)	251	26.9	269	22.8	132	22.8	139	17.6	206	28.8	177	17.6	126	24.4	139	21.8
Overweight (25- <30 kg/m^2^)	386	41.4	347	29.4	231	39.9	257	32.5	264	36.8	304	30.3	212	40.9	218	34.2
Obese (≥30 kg/m^2^)	295	31.7	565	47.8	216	37.3	394	49.9	246	34.4	522	52.1	180	34.7	280	44.0
**Serum Cholesterol**																
Normal (<5.18 mmol/L)	627	67.3	849	71.9	290	50.1	417	52.8	332	46.4	513	51.1	354	70.8	413	68.8
Moderate hypercholesterolemia^§^	212	22.7	223	18.9	194	33.5	245	31.0	260	36.3	336	33.5	121	24.2	147	24.5
High hypercholesterolemia^¶^	93	10.0	109	9.2	95	16.4	128	16.2	124	17.3	154	15.4	25	5.0	40	6.7
**Education Level**																
Up to grade VIII	247	26.5	373	31.6	156	26.9	277	35.1	158	22.1	306	30.5	116	22.4	201	31.6
High school/Diploma	423	45.4	458	38.8	264	45.6	312	39.5	314	43.8	408	40.7	225	43.4	272	42.7
≥ Undergraduate	262	28.1	350	29.6	159	27.5	201	25.4	244	34.1	289	28.8	177	34.2	164	25.7
**Exercise**																
No	566	60.7	843	71.4	380	65.6	588	74.4	397	55.4	756	75.4	300	57.9	515	80.8
Yes	366	39.3	338	28.6	199	34.4	202	25.6	319	44.6	247	24.6	218	42.1	122	19.2
**Smoking Status**																
No	741	79.5	1116	94.5	474	81.9	781	98.9	496	69.3	989	98.6	356	68.7	631	99.1
Yes	191	20.5	65	5.5	105	18.1	09	1.1	220	30.7	14	1.4	162	31.3	06	0.9

^§^Serum cholesterol ≥5.18 but ≤ 6.22 mmol/L. ^¶^ Serum cholesterol >6.22 mmol/L.

Unadjusted prevalence of IFG increased slightly with time until 2004–05 in males and then decreased to 6.0%, a 61% decrease which was significant (*P*<0.05), in 2008–09 (Table 2). In females, prevalence of IFG decreased with time, except for 2006–07, with a significant (P<0.05) decline to 5.3%, a 57% decrease, in 2008–09. During the 8 years (2002–09), the prevalence of IFG in males and females decreased by 7.4% and 6.8%, respectively. In both genders, prevalence of diabetes also decreased with time period. During the 8 year period (2002–09), the prevalence of diabetes in males and females fell by 9.8% and 8.9%, respectively. Prevalence of both IFG and diabetes were higher in males than females throughout the study period. However, for IFG the difference was statistically significant (*P*<0.05) only in 2004–05 and for diabetes, it was significant (*P*<0.05) in 2002–03 and 2008–09.

**Table 2:** Prevalence of IFG and diabetes among Kuwaiti males and females by study period, socio-demographic, biological and lifestyle factors

	**Male (n=2745)**			**Female (n=3611)**		
**Variable**	**n**	IFG^*^	Diabetes**	**n**	**IFG**	**Diabetes**
**Study Period**						
2002-03	932	13.4^b1^	19.1^b1^	1181	12.1^c1^	14.9^c2^
2004-05	579	15.4^b1^	16.8^b1^	790	9.2^b2^	13.8^bc1^
2006-07	716	14.0^b1^	12.2^a1^	1003	12.4^c1^	11.3^b1^
2008-09	518	6.0^a1^	9.3^a1^	637	5.3^a1^	6.0^a1^
**Age Group (Year)**						
20-29	930	9.1^a1^	4.4^a1^	1246	8.2^a1^	3.4^a1^
30-39	660	11.1^a1^	13.0^b1^	857	8.9^a1^	6.2^b2^
40-49	635	15.1^b1^	16.9^c1^	842	11.9^b1^	13.9^c1^
50-59	345	16.8^b1^	30.4^d1^	508	14.0^b1^	30.9^d1^
60-69	175	18.9^b1^	40.6^e1^	158	15.8^b1^	42.4^e1^
**BMI Status**						
Normal (<25 kg/m^2^)	715	9.5^a1^	8.5^a1^	724	6.8^a1^	3.6^a2^
Overweight (25- <30 kg/m^2^)	1093	13.5^b1^	14.8^b1^	1126	9.6^b2^	9.1^b2^
Obese (≥30 kg/m^2^)	937	13.8^b1^	20.0^c1^	1761	12.3^c1^	17.5^c1^
**Serum Cholesterol**						
Normal (<5.18 mmol/L)	1603	11.4^a1^	12.7^a1^	2192	9.8^a1^	10.2^a2^
Moderate hypercholesterolemia^§^	787	13.7^ab1^	16.0^b1^	951	11.8^a1^	13.5^b1^
High hypercholesterolemia^¶^	337	15.7^b1^	23.7^c1^	431	9.7^a2^	19.3^c1^
**Education Level**						
Up to grade VIII	667	14.5^a1^	21.4^b1^	1157	13.7^c1^	21.2^b1^
High school & diploma	1226	12.1^a1^	13.5^a1^	1450	9.8^b2^	8.6^a2^
Undergraduate and above	842	11.8^a1^	11.9^a1^	1004	7.4^a2^	6.6^a2^
**Exercise**						
No	1643	13.0^a1^	16.9^b1^	2702	10.4^a2^	12.7^b2^
Yes	1102	11.9^a1^	12.1^a1^	909	10.3^a1^	10.2^a1^
**Smoking Status**						
No	2067	13.8^b1^	15.3^a1^	3517	10.5^b2^	11.9^a2^
Yes	678	8.7^a1^	13.7^a1^	94	4.3^a1^	17.0^a1^

n= total number of subjects.

^*^Fasting blood glucose level > 6.1 but <7.0 mmol/L; ^**^Fasting blood glucose >7.0 mmol/L.

^§^Serum cholesterol >5.18 but < 6.22 mmol/L; ^¶^Serum cholesterol >6.22 mmol/L.

^a,b,c^Prevalence value within a column with unlike superscript letters were significantly different (*P*<0.05) using Z –test.

^1,2,3^Prevalence values across the rows for each listed variable with unlike numbers were significantly different (P<0.05) using Z-test.

IFG and diabetes prevalences were also affected by age (Table 2). IFG prevalence in males increased significantly (*P*<0.05) from 9.1% in the 2^nd^ age decade to 18.9% in the 6^th^. Diabetes prevalence also increased significantly with increasing age decades, reaching a maximum (40.6%) in the 6^th^ decade. In females, IFG prevalence increased significantly (*P*<0.05) from 8.2% in the 2^nd^ decade to 15.8% in the 6^th^ decade. The diabetes prevalence also increased with increasing age decades, reaching a maximum (42.4%) in the 6^th^ decade. There were no significant differences in the prevalences of IFG and diabetes between males and females throughout the age decades, except for diabetes in females in the 2^nd^ decades.

In both genders, the prevalence of IFG and diabetes increased significantly with overweight and obesity, and decreased with level of education, except for IFG in males (Table 2). Diabetes prevalence increased significantly with TC levels and decreased with any form of exercise. Both male and female smokers had lower prevalence of IFG with no significant changes in the prevalence of diabetes.

Regression coefficients (B) indicated an overall 0.125 mmol/l yearly decrease in FBG (1 mmol/L for the entire study period). Males had a greater decrease than females (1.12 vs 0.92 mmol/L); they also showed an increase in the 2002–2003 time segment followed by a marked decrease in 2006–2007 while females showed a significant time trend only within 2008–2009 (Table 3). Both males and females showed the largest decrease in the 2^nd^ half of the study accounting for the majority of the overall decrease (1.13 mmol/L for males and 0.87 mmol/l for the 4 year period). The largest yearly decreases for any confounder were for age >40y, particularly for males. Males with less education or who were obese also showed a greater change than the gender as a whole.

**Table 3:** Linear regression analysis for predicting glucose levels for time within each study period segment, age decade, BMI and education category

	**Male**			**Female**		
	**N**	**B ( mmol/L)**	**P-Value**	**N**	**B ( mmol/L)**	**P-Value**
**Study period**						
2002-3	931	0.685	0.0001	1180	−0.115	0.456
2004-5	578	−0.133	0.486	789	0.040	0.704
2006-7	715	−0.513	0.001	1002	−0.128	0.240
2008-9	499	−0.227	0.173	636	−0.200	0.048
2002-5	1511	−0.082	0.207	1970	−0.158	0.002
2006-9	1215	−0.282	0.0001	1639	−0.213	0.0001
Total	2726	−0.140	0.0001	3610	−0.115	0.0001
**Age Decades**						
20s & 30s	1582	−0.087	0.0001	2102	−0.102	0.0001
40s-60s	1143	−0.228	0.0001	1507	−0.119	0.0001
**BMI Category (kg/m**^**2**^**)**						
<30	1798	−0.119	0.0001	1850	−0.122	0.0001
≥30	927	−0.171	0.0001	1759	−0.098	0.0001
**Education Category**						
≤ High school or diploma	1890	−0.182	0.0001	2606	−0.114	0.0001
Undergraduate and above	835	−0.096	0.002	1003	−0.108	0.0001

B is the slope for regression line. Each variable was adjusted for others.

Logistic regression analysis was used to determine temporal trends in prevalence of IFG and diabetes for 2 year spans compared with 2002–03 using both unadjusted model and after adjusting for age, BMI, HC and education levels for males and females separately. Although unadjusted trends in prevalence of IFG and diabetes in both males and females were similar to that of adjusted trends, there were slightly higher OR in each study period. Therefore, we presented the adjusted OR (Table 4). Compared with 2002–03, the odds ratio (OR) of IFG in males decreased with time, and became significant (OR=0.32; 95% CI: 0.21-0.49) for 2008–09. In females, the odds of IFG decreased significantly with time period, except in 2006–07. There was a gradual significant decrease in odds of diabetes with time period in both males and females. Compared with 2002–03, the OR for diabetes in males was 0.34 (95% CI: 0.24-0.49) and in females was 0.33 (95% CI: 0.22-0.50) in 2008–09.

**Table 4:** Odds Ratio (OR)* for IFG and diabetes associated with study period among Kuwaiti males and females

	IFG**				Diabetes***			
**Study Period**	**OR**	**95% CI**		***P*-value**	**OR**	**95% CI**		***P*-value**
**Male**								
2002-03	1.00				1.00			
2004-05	0.99	0.72	1.35	0.93	0.65	0.48	0.89	0.006
2006-07	0.88	0.65	1.18	0.36	0.52	0.39	0.71	0.001
2008-09	0.32	0.21	0.49	0.001	0.34	0.24	0.49	0.001
**Female**								
2002-03	1.00				1.00			
2004-05	0.70	0.52	0.96	0.024	0.78	0.58	1.04	0.090
2006-07	0.98	0.75	1.28	0.867	0.76	0.57	1.00	0.050
2008-09	0.32	0.21	0.49	0.001	0.33	0.22	0.49	0.001

*Adjusted for age, education, BMI and serum cholesterol level.

**Fasting blood glucose level ≥ 6.1 but <7.0 mmol/L. ***Fasting blood glucose ≥7.0 mmol/L.

A second logistic regression model was carried out to determine the contribution of all the investigated risk factors of IFG and diabetes. When compared with the 2^nd^ age decade, the odds of having IFG increased significantly from the 4^th^ age decade until the 6^th^ decade in both males and females (Table 5). Both overweight and obese males and females were significantly more likely to have IFG than those with normal BMI. Only in females did the odds of IFG decrease significantly with increased level of education. Compared with non-smokers, the odds of IFG decreased significantly among male and female smokers. No significant association was found for either hypercholesterolemia or exercise in either gender.

**Table 5:** Odds Ratio (OR) for IGT and diabetes associated with selected socio-demographic, biological and lifestyle factors among Kuwaiti males and females

	IFG*								Diabetes**							
	**Male**				**Female**				**Male**				**Female**			
	**OR**	**95% CI**		**P-value**	**OR**	**95% CI**		**P-value**	**OR**	**95% CI**		**P-value**	**OR**	**95% CI**		**P-value**
**Age Decade (Year)**																
20-29	1.00				1.00				1.00				1.00			
30-39	1.23	0.87	1.72	0.241	0.96	0.69	1.32	0.783	2.94	1.98	4.37	0.001	1.51	0.99	2.31	0.059
40-49	1.71	1.23	2.39	0.002	1.37	1.00	1.88	0.050	4.22	2.85	6.26	0.001	3.53	2.40	5.18	0.001
50-59	2.41	1.63	3.55	0.001	2.01	1.39	2.90	0.001	9.48	6.25	14.37	0.001	9.08	6.10	13.52	0.001
60-69	3.38	2.04	5.60	0.001	2.57	1.48	4.45	0.001	16.27	10.02	26.42	0.001	14.85	8.98	24.57	0.001
**BMI Status**																
Normal (<25 kg/m^2^)	1.00				1.00				1.00				1.00			
Overweight (25- <30 kg/m^2^)	1.39	1.02	1.91	0.040	1.40	0.97	2.02	0.070	1.43	1.02	2.00	0.038	1.82	1.14	2.89	0.012
Obese (≥30 kg/m^2^)	1.59	1.15	2.19	0.006	1.77	1.25	2.51	0.001	2.14	1.53	2.98	0.001	2.88	1.85	4.67	0.001
**Serum Cholesterol Status**																
Normal (<5.18 mmol/L)	1.00				1.00				1.00				1.00			
Moderate hypercholesterolemia^§^	1.07	0.82	1.40	0.634	1.04	0.81	1.34	0.764	0.91	0.70	1.19	0.507	0.91	0.71	1.18	0.491
High hypercholesterolemia^¶^	1.28	0.90	1.82	0.173	0.84	0.58	1.21	0.340	1.32	0.95	1.83	0.095	1.22	0.89	1.66	0.217
**Education Level**																
Up to Grade VIII	1.00				1.00				1.00				1.00			
High school & diploma	0.85	0.63	1.14	0.278	0.75	0.58	0.98	0.038	0.79	0.60	1.04	0.095	0.73	0.56	0.95	0.021
Undergraduate and above	0.79	0.57	1.09	0.158	0.58	0.42	0.80	0.001	0.68	0.50	0.92	0.014	0.65	0.47	0.90	0.009
**Exercise**																
No	1.00				1.00				1.00				1.00			
Yes	0.97	0.76	1.23	0.792	1.01	0.78	1.30	0.95	0.82	0.64	1.04	0.105	0.84	0.65	1.10	0.206
**Smoking**																
No	1.00				1.00				1.00				1.00			
Yes	0.68	0.50	0.93	0.015	0.36	0.13	1.00	0.050	1.20	0.91	1.59	0.200	1.08	0.58	2.03	0.803

^*^Fasting blood glucose is >6.1 but <7.0 mmol/L. **Fasting blood glucose ≥7.0 mmol/L.

^§^Serum cholesterol is ≥5.18 but ≤ 6.22 mmol/L. ^¶^Serum cholesterol is >6.22 mmol/L.

Similarly, the odds of diabetes increased significantly with age from the 2^nd^ until the 6^th^ decade in both genders. Both overweight and obese males and females were significantly more likely to be diabetic than those with normal BMI. The odds of diabetes decreased significantly with increased level of education in both genders. No significant association was found with HC, exercise or smoking in either gender (Table 5).

## Discussion

Temporal changes in unadjusted prevalence of IFG and diabetes among Kuwaiti adults in the 8 years since 2002 show a significant decrease in both males and females. When adjusted for age, BMI, HC and education level, logistic regression analysis showed that, compared with 2002–03, both males and females remained significantly less likely to be diabetic and/or to have IFG in 2008–09 and thus confirmed the declining trends in both IFG and Diabetes among Kuwaiti adults. Time trend data for the region that can be used for comparison: a study among Iranians showed an increased trend for diabetes from 2005 to 2007 [16], as did data from Oman for 1991–2000, Saudi Arabia for 1993–2000, and UAE for 1995–2000 [8]. However, these studies represented earlier data collection period comparable to the earlier part of our study except Iranian study (2005–07).

The overall crude prevalences of IFG and diabetes were significantly higher in males than females. When the data were stratified by study periods, the prevalence of IFG in males was significantly higher than females only in 2004–05, and for diabetes, it was higher in males than in females in 2002–03 and 2008–09. The results of linear regression showed a greater decrease over time in males and closer values between the genders in 2008–2009. When logistic regression was carried out with combined genders, after adjusting for various factors, females were less likely to have IFG (OR: 0.65; 95% CI: 0.55-0.77) and/or diabetes (OR: 0.63; 95% CI: 0.53-0.75) than males. A similar gender difference in diabetes has recently been reported in Kuwaitis [12], though a lower prevalence of diabetes was reported in Kuwaiti males in an earlier study [17]. Studies from other countries in the region also reported mixed results. Men had higher prevalences of diabetes in Saudi Arabia [18], Oman [19], Yemen [20] and Jordan [21], while women had higher prevalences in Iran [16], UAE [22] and Bahrain [23]. These variabilities between countries in diabetes with gender may reflect the variation in other factors that can influence diabetes including patient identification. Further, in the present study we found a significant interaction of education level and gender with the prevalence of IFG and diabetes. Thus, it is also possible that some of the “confounders” that we adjusted for in our analysis altered the apparent gender differences.

In the present study we also explored the association of selected socio-demographic (age, education), biological (BMI and serum TC) and lifestyle (exercise and smoking) factors. As observed in earlier studies among Kuwaitis [12,17], and as would be expected, age was found to be an important predictor of IFG and diabetes prevalence for both genders in the present study. The odds of IFG and diabetes increased with age until the 6^th^ decade in both genders. A similar age related change in diabetes has also been reported in other studies in the Arab Gulf region [16,18,22,24].

In the present study, both overweight and obese males and females were more likely to have IFG and diabetes which are in accordance with the findings from other countries in the region [9,18,25-28] where a consistently positive association of overweight and obesity with diabetes is demonstrated. It is known that adipocytes (fat cells) secrete a number of adipocyte hormones and adipokines, which may in turn increase the risk of diabetes via several pathways such as increasing insulin resistance [29].

Unadjusted prevalence of diabetes was significantly higher in both genders with moderate-HC and high-HC than in individuals with normal cholesterol level. After adjusting for confounding factors in logistic regression, the OR for diabetes in males (OR: 1.32; 95% CI: 0.95-1.83) and females (OR: 1.22; 95% CI: 0.89-1.66) were higher in those with high-HC than the subjects with normal cholesterol level, however they did not reach the level of significance.

More highly educated females were significantly less likely to have IFG or diabetes in our study, but only highly educated (undergraduate or above) males were less likely to be diabetes. An earlier study in Kuwaiti adults also showed that higher education level was associated with a significant reduction in blood glucose levels [30]. Furthermore, a lower education level has been found to be associated increased likelihood of diabetes among Bahrainis [23], Omanis [24] and Iranians [31]. It is possible that those who were more highly educated may have greater awareness of the risk factors of hyperglycemia and diabetes, and thus more likely to practice more effective preventing measures. However, higher education had no significant impact on IFG prevalence in Kuwaiti males implying that they are either less aware of the risk factors of hyperglycemia.

The prevalence rates indicate that both males and females who were exercising were possibly less likely to be diabetic than those who were not exercising. However, the results (odds ratio) were not statistically significant when adjusted for other confounders including BMI supporting excess body weight as the primary risk factor. Kuwaitis who participate in even moderate exercise have been shown to have delayed weight gain with age [32]. In a recent study we have also reported that the Kuwaiti males who exercised were significantly less likely to be obese [33]. Since we do not have any information on the duration and intensity of the exercise it is difficult interpret the present findings.

We found that both males and females who were smokers were significantly less likely to have IGF than non-smokers even when adjusted for age and BMI, though no significant association between smoking and diabetes was observed. Studies from UAE [28] and Qatar [25] showed that smokers were more likely to be diabetic than non-smokers, while a longitudinal study in Iranian adults found no association with smoking and incidence of diabetes [31]. It is important to note that most prospective studies have shown higher risk of diabetes for smokers, especially those who smoke more than 1 packet per day [34]. Unfortunately, in the present study we do not have information on the frequency/intensity and duration of the smoking. Further, it should be noted that smoking may not be a protective for IFG. Since the study is cross sectional, no cause and effect could be determined.

This study has a number of limitations. The prevalences of IFG and diabetes were estimated by single measurement of blood glucose using Accutrend GCT, which could introduce some errors. However, given that the survey included a relatively large population-based sample it is unlikely that the true prevalence will be different than that we have reported here. Further, in the present study those with known diabetes (those taking oral agents or insulin) were excluded. It is possible that this number has been increasing over time with more attention to diabetes and its treatment by Physicians, and thus our estimation of diabetes might have underestimated the prevalence in recent years, but the prevalence is still alarmingly high. Given its cross-sectional nature no causal relationship of socio-demographic and lifestyle factors with IFG and diabetes prevalence can be established. There was no information on the frequency/intensity and duration of the smoking and exercise pattern. More carefully designed studies that capture lifetime exercise, smoking and food intakes are needed to explore these potential associations between lifestyle factors and hyperglycemia. While efforts were made to obtain representative samples, the KNSS uses convenience sampling and thus the results of the present may not be representative of the wider population. Nevertheless, the main strength of this study is that it includes a relatively large population-based sample so it is likely that the true situation will not be different, on the whole, than reported here.

## Conclusion

A decrease in the prevalence of IFG and diabetes was observed between 2002–03 and 2008–09 even when data was adjusted for known variables such as age and obesity and the suspected confounders education level, smoking and exercise. Continued monitoring is needed to determine if these decreases are persistent, as well as more detailed assessments of exercise and other confounders to determine the magnitude and duration needed to be protective. Lastly inclusion of those undergoing treatment for diabetes would facilitate evaluation of existing identification and treatment programs, and of the total prevalence of diabetes

## Competing interests

The authors declare that they have no competing interests.

## Authors’ contributions

FA and CW took the lead in the study concept, analysis and wrote the manuscript. MAA provided guidance on data collection and contributed to writing. AA contributed in writing the manuscript. PP was responsible for data collection, management and cleaning. All authors read and approved the final manuscript.

## Pre-publication history

The pre-publication history for this paper can be accessed here:

http://www.biomedcentral.com/1471-2458/13/103/prepub
